# Notes and News

**Published:** 1895-01-05

**Authors:** 


### NOTES AND NEWS.

COLLECTED AND COMMUNICATED.

KING'S NORTON is to have a new Union Infirmary. Mr. Rowbotham, of Birmingham, has undertaken the work, his estimate amounting to £17,987.

CHOLERA has appeared, in isolated cases, in South America, at Rosario, La Plata, San Nicholas, and Cordoba.

THE Health Department of the United States Government have decided to prosecute the vendors of spurious antitoxin.

THE Royal Hospital at Belfast has found a generous friend in Mrs. Dunville, who has furnished the women's operation ward at the institution in a most comfortable manner, and provided all necessary appliances of the most approved design.

WE regret that an error crept into our appeal on behalf of the German Hospital, crediting that institution with 20 instead of 120 beds, but as we stated that 1,457 patients were received within the wards in one year, the work of the hospital was clearly apparent.

THE Surgeon-General of the United States Navy, in making his annual report, attributes the unusually low percentage of mortality from pneumonia to the uniform treatment adopted, which consisted of a hypodermic injection of $\frac{1}{30}$ to $\frac{1}{35}$ grain of sulphate of strychnine every three or four hours, together with a very free use of alcohol.

SANITARY regulations appear to be well carried out in the ships of the United States Navy, and to this fact is attributed the immunity of American ships from visitations of yellow fever in infected ports. So successful have the precautions proved to be when adopted, that measures are being taken to ensure their complete and universal adoption throughout the navy. Perhaps we may safely conjecture that sanitary measures which will keep yellow fever at bay, will not be altogether without effect in reducing the mortality from pneumonia.

FEMALE medical students at Brussels have been signally successful at the recently held examinations. All three candidates for the degree of doctor passed with much credit, two of them being greatly distinguished. The success is all the more marked as of the male students five only passed at all, and none passed with distinction.

"GUY'S HOSPITAL MAGAZINE" for December 27th is rendered especially attractive by a beautifully-executed presentation plate, representing Thomas Guy, the founder of the hospital, conferring with the architects upon the original plans of the building. The painting from which the plate is taken is by C. W. Cope, R.A., and belongs to the hospital.

PROFESSOR BEHRING, who is staying at Paris, is receiving quite an ovation in medical circles. M. Dupuy has informed him that the Government intends to bestow upon him the Cross of the Legion of Honour. Professor Behring has stated that he believes that when the use of anti-toxin is universally established, deaths from diphtheria will average less than 5 per cent.

THE sick children in the North-West London Hospital are wishing those who have contributed towards the institution which shelters them a Merry Christmas and a Happy New Year through the medium of a charmingly-executed reproduction of a picture by Alfred Dobson. We hope the appealing little face in the picture will move the hearts of the public towards the assistance of the class of little ones it represents.

WHATEVER the final verdict for or against the efficacy of the anti-toxin method of treatment for diphtheria, its rapidly spreading adoption throughout the civilised world will supply ample statistics for consideration. Apart from English enterprise, in New York the Press is subscribing to supply the poor. In France the east portion of the country is to have an institute to itself. At Amsterdam the Press and various societies are subscri bing towards the preparation of serum, by Professor Forster. And at Barcelona, in Spain, the Municipal Laboratory has commenced operations.

THE resignation by Sir Henry Acland of the Regius Professorship of Physic at Oxford will deprive the University of one of its most striking features. It may be said that Professor Acland "made" Medicine at Oxford. Nevertheless, it is not as a maker of a generation of doctors that he should be known now and for all time, but rather as a guide and a teacher to a higher attainment. First, scientific thinkers and observers; then students in the great schools of disease; later, workers in the field of suffering humanity. That was his standard for the education of the physician, and for the establishment of such a standard in the University of Oxford has he ever laboured. The courtly gentleman, the cultivated student of art and letters, the kindly friend and counsellor, the skilful physician, he himself has offered an example of all he taught, and though in respect to the University of Oxford we must speak of him in the past tense, the effect of such an example must continue to work for good in the large sphere of his private life.

